# Suppression of mitochondrial oxygen metabolism mediated by the transcription factor HIF-1 alleviates propofol-induced cell toxicity

**DOI:** 10.1038/s41598-018-27220-8

**Published:** 2018-06-12

**Authors:** Chisato Sumi, Akihisa Okamoto, Hiromasa Tanaka, Munenori Kusunoki, Tomohiro Shoji, Takeo Uba, Takehiko Adachi, Teppei Iwai, Kenichiro Nishi, Hiroshi Harada, Hidemasa Bono, Yoshiyuki Matsuo, Kiichi Hirota

**Affiliations:** 1 Department of Anesthesiology, Kansai Medical University, Hirakata, Japan; 2 Department of Human Stress Response Science, Institute of Biomedical Science, Kansai Medical University, Hirakata, Japan; 3 Department of Anesthesiology, Tazuke Kofukai Medical Research Institute, Osaka, Japan; 4 Laboratory of Cancer Cell Biology, Radiation Biology Center, Kyoto University, Kyoto, Japan; 5 Precursory Research for Embryonic Science and Technology (PRESTO), Japan Science and Technology Agency (JST), Saitama, Japan; 6 Database Center for Life Science (DBCLS), Research Organization of Information and Systems (ROIS), Mishima, Japan

## Abstract

A line of studies strongly suggest that the intravenous anesthetic, propofol, suppresses mitochondrial oxygen metabolism. It is also indicated that propofol induces the cell death in a reactive oxygen species (ROS)-dependent manner. Because hypoxia-inducible factor 1 (HIF-1) is a transcription factor which is involved in cellular metabolic reprogramming by modulating gene expressions of enzymes including glycolysis pathway and oxygen utilization of mitochondria, we examined the functional role of HIF-1 activity in propofol-induced cell death. The role of HIF-1 activity on oxygen and energy metabolisms and propofol-induced cell death and caspase activity was examined in renal cell-derived RCC4 cells: RCC4-EV cells which lack von Hippel-Lindau protein (VHL) protein expression and RCC4-VHL cells, which express exogenous VHL, and in neuronal SH-SY5Y cells. It was demonstrated that HIF-1 is involved in suppressing oxygen consumption and facilitating glycolysis in cells and that the resistance to propofol-induced cell death was established in a HIF-1 activation-dependent manner. It was also demonstrated that HIF-1 activation by treatment with HIFα-hydroxylase inhibitors such as n-propyl gallate and dimethyloxaloylglycine, alleviated the toxic effects of propofol. Thus, the resistance to propofol toxicity was conferred by HIF-1 activation by not only genetic deletion of VHL but also exposure to HIFα-hydroxylase inhibitors.

## Introduction

Propofol (2,6-diisopropylphenol) is used for anesthesia in operating theaters and for sedation in intensive care units around the world^1^. Propofol is recognized as a safe and effective drug. However, it can cause a rare but severe complication, especially in patients receiving high doses for prolonged periods. This syndrome is known as propofol infusion syndrome^2–4^. Although the morbidity of the syndrome is approximately 1%, even among critically ill patients, mortality is >50%^4^. Thus, this syndrome is one of the most significant issues to be addressed in the field of critical care medicine.

We previously demonstrated that clinically relevant concentrations of propofol used within a clinically relevant exposure time suppressed mitochondrial function, induced the generation of reactive oxygen species (ROS), and caused metabolic reprogramming from oxidative phosphorylation (OXPHOS) to glycolysis by targeting mitochondrial complexes I, II and III^5^. In addition, we showed that the local anesthetic, lidocaine, induced ROS generation, which was attenuated by forced activation of hypoxia-inducible factor 1 (HIF-1)^6^.

HIF-1 is a transcription factor that functions as a master regulator of oxygen homeostasis^7,8^. This heterodimeric protein is composed of a constitutively expressed HIF-1β subunit and an O_2_-regulated HIF-1α subunit under normoxic conditions. HIF-1α is subjected to prolyl hydroxylation by oxygenases, which utilize O_2_ as a substrate^9^. Hydroxylation modification is required for binding of the von Hippel-Lindau (VHL) protein, which targets HIF-1α for ubiquitination and proteasomal degradation^10^. Thus, even under normoxic conditions, hydroxylase inhibitors such as dimethyloxaloylglycine (DMOG) and n-propyl gallate (nPG) can activate HIF-1^6,11–13^. Accordingly, the genetic ablation of VHL also activate HIF-1 even under normoxic conditions^10^. Intriguingly, mitochondrial function can be regulated by HIF-1^14–16^. OXPHOS is regulated by several mechanisms, including substrate availability. Pyruvate is one of the substrates determining OXPHOS and electron transport in mitochondria. Pyruvate is converted to acetyl-CoA by the pyruvate dehydrogenase complex; this is regulated by pyruvate dehydrogenase kinases, the expression of which is regulated by HIF-1^17^. Thus, HIF-1 actively regulates the oxygen metabolism of the cells by coordinating mitochondrial function. Thus the efficient use of available oxygen explain how HIF-1 activation suppresses the generation of harmful byproducts such as ROS^17–19^.

In this study, we investigate the role of HIF activation on propofol-induced apoptosis in renal cell-derived RCC4 cells and neuronal SH-SY5 cells, and demonstrate that activation of HIF-1 ameliorates propofol toxicity by modulating mitochondrial function and ROS generation.

## Results

### RCC4-EV cells are resistant to propofol-induced cell

**Figure 1:**
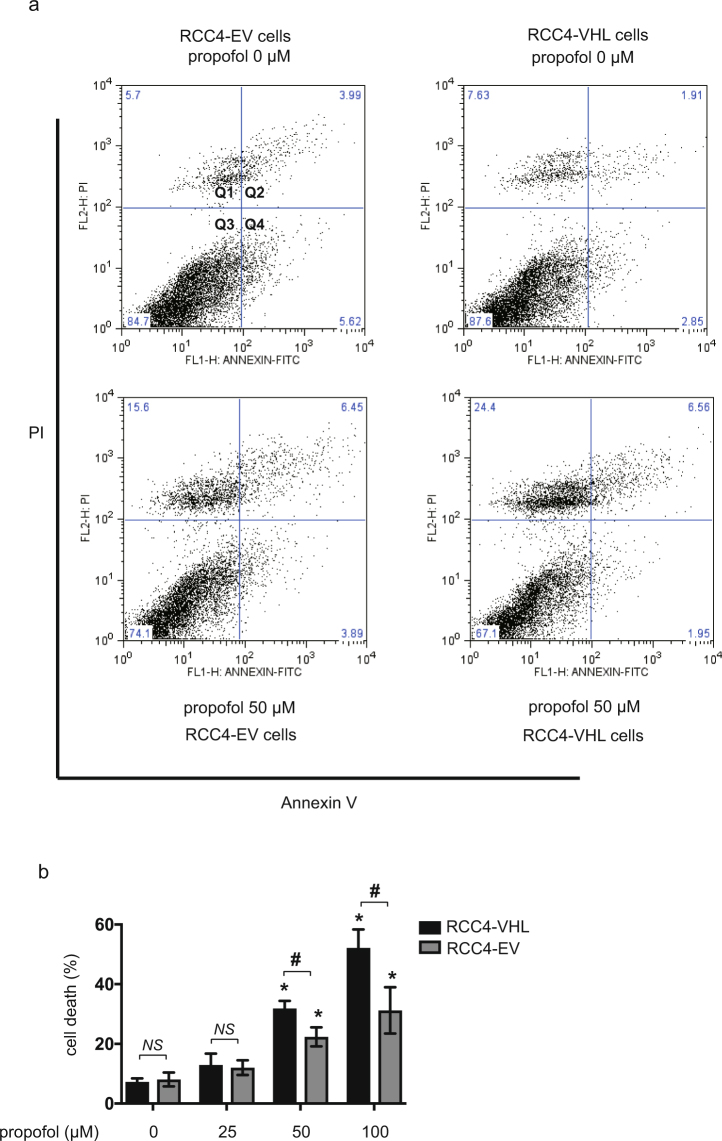
RCC4-EV cells are more resistant to propofol-induced cell injury than RCC4-VHL cells. RCC4-VHL and RCC4-EV cells were exposed to the indicated propofol concentrations for 6 h. (**a**) Cells were harvested and cell death was detected by flow cytometry. The ratio of annexin V- and/or PI-positive positive cells [(Q1 + Q2 + Q4)/(Q1 + Q2 + Q3 + Q4)] was used to calculate the percentage of dead cells. (**b**) The cell death are shown for each treatment group (n = 3). *p < 0.05, as compared to the control cell population (no treatment); ^#^*p* < 0.05, for the indicated comparison.

**Figure 2:**
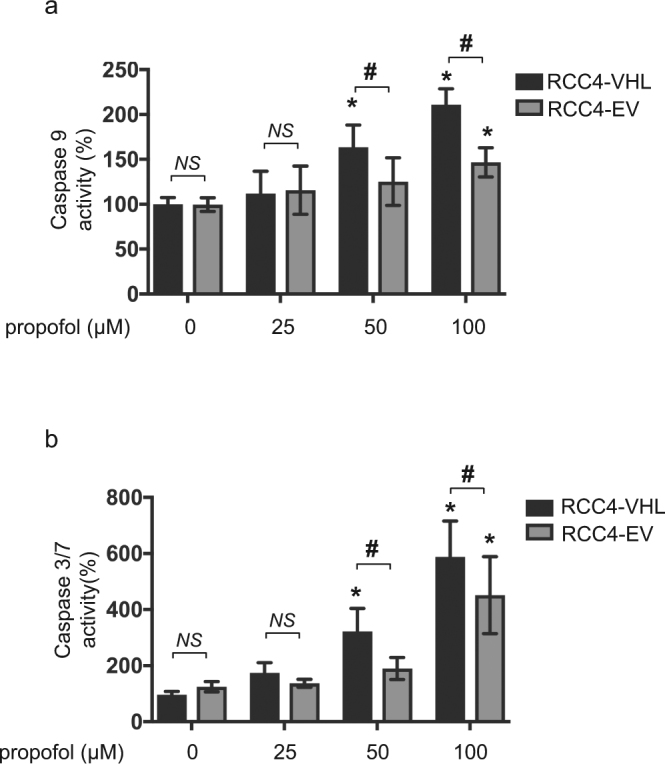
Propofol-induced caspases activation is attenuated in RCC4-EV cells than RCC4-VHL cells. RCC4-VHL and RCC4-EV cells were exposed to the indicated propofol concentrations for 6 h. The levels of (**a**) caspase 9 (n = 5) and (**b**) caspase 3/7 (n = 5) activity are shown for each treatment group. Differences between results were evaluated by two-way ANOVA followed by Dunnett’s test for multiple comparisons; *p < 0.05, as compared to the control cell population (no treatment); ^#^*p* < 0.05, for the indicated comparison.

It is reported that RCC4-EV cells do not express VHL protein and they therefore constitutively express HIF-1α, a regulatory subunit of HIF-1 even under normoxic (20% O_2_) conditions^10^. In contrast, RCC4-VHL cells express exogenous VHL protein, and HIF-1α expression is therefore regulated in an oxygen tension-dependent manner. To determine the dose- and time-response relationship between propofol treatment and cell death, we treated RCC4-EV and RCC4-VHL cells with the indicated doses of propofol for 6 h (Fig. 1) under 20% O_2_ conditions. Effect of propofol on the cell death of RCC4-EV cells and RCC4-VHL cells were examined by flow cytometry with PI and FITC-conjugated annexin V staining (Fig. 1a). The cell death following treatment with 50 µM propofol for 6 h was significantly suppressed in RCC4-EV cells compared to RCC4-VHL cells (Fig. 1b). Next, we investigated the caspases activations under propofol treatment. Concentrations > 50 µM propofol induced caspase 9 activation within 6 h. 50 µM and 100 µM propofol induced caspase 9 activation significantly differentially in RCC4-EV cells and RCC4-VHL cells (Fig. 2a). Next, caspase 3/7 activity was evaluated following exposure of both RCC4-VHL and RCC4-EV cells to propofol for 6 h. Similarly to caspase 9, a significant difference in caspase 3/7 activity was detected between RCC4-EV cells and RCC4-VHL cells following propofol treatment (Fig. 2b).

### HIF-1 is activated in RCC4-EV cells under 20% O_2_ conditions

**Figure 3:**
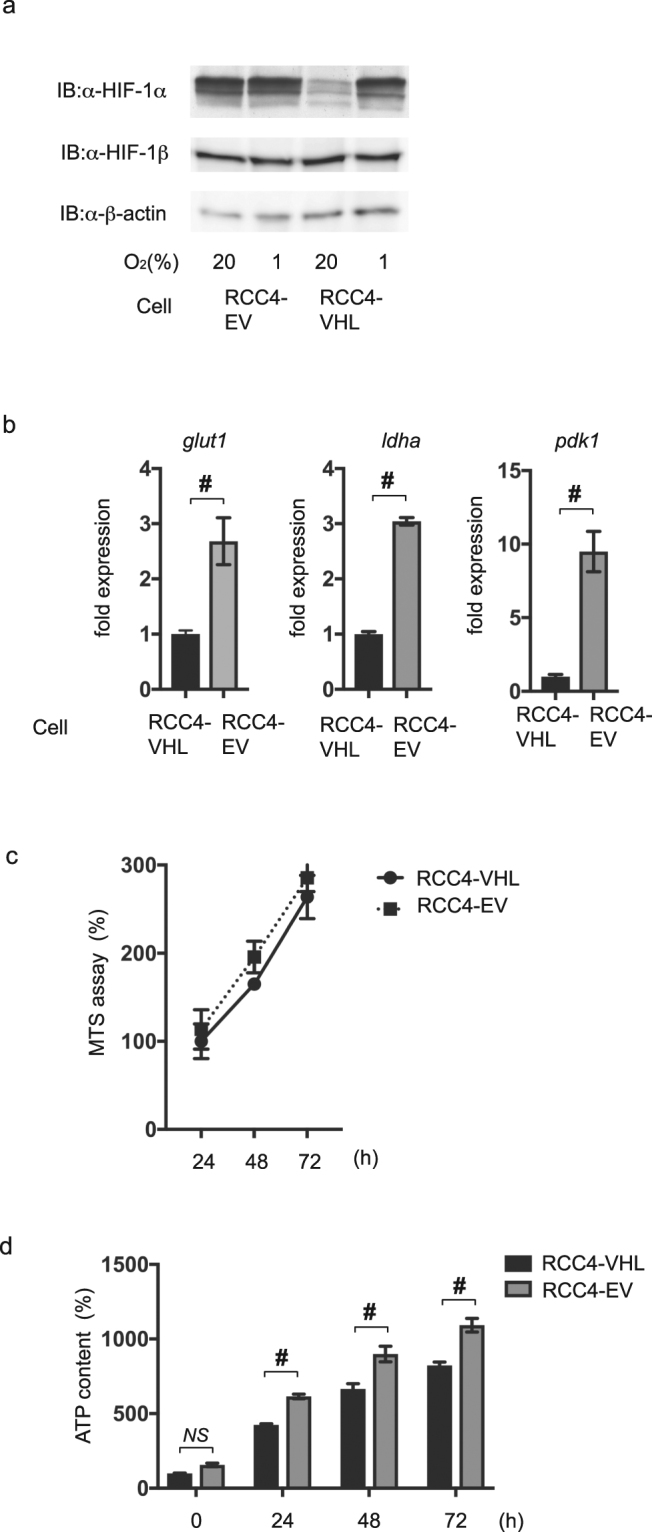
HIF-1 is activated in RCC4-EV cells under normoxic conditions. (**a**) 35 µg whole-cell lysates of RCC4-EV and RCC4-VHL cells exposed to either 20% O_2_ or 1% O_2_ were immunoblotted (IB) using primary antibodies raised against the indicated proteins. (**b**) RCC4-EV and RCC4-VHL cells were cultured with 20% O_2_ prior to analysis of the indicated mRNA levels using semi-quantitative *q*RT-PCR. Fold expression was calculated based on expression in RCC4-VHL cells incubated with 20% O_2_. Data presented are expressed as mean ± SD; ^#^*p* < 0.05, for the indicated comparison. (**c**) RCC4-VHL and RCC4-EV cells were cultured for the indicated time-periods prior to cell viability evaluation by MTS assay (n = 3). (**d**) RCC4-VHL and RCC4-EV cells were cultured for the indicated time-periods prior to determination of the cellular ATP level. Differences between results were evaluated by *t*-test; ^#^*p* < 0.05 for the indicated comparison.

HIF-1 activation was investigated in RCC4-VHL and RCC4-EV cells cultured under normoxic (20% O_2_) and hypoxic (1% O_2_) conditions. The protein expression levels of HIF-1α and HIF-1β were assayed by immunoblot analysis (Fig. 3a). Consistent with a previous report^10^, HIF-1α was constitutively expressed in RCC4-EV cells, even in the presence of 20% O_2_, at levels comparable to those observed in RCC4-VHL cells cultured in the presence of 1% O_2_. HIF-1β expression was stable in both cell types under both O_2_ levels. Consistent with HIF-1α protein expression findings, the mRNA levels of downstream genes including glucose transporter 1 (*glut1*), lactate dehydrogenase A (*ldha*) and pyruvate dehydrogenase kinase 1(*pdk1*) were higher in RCC4-EV cells than in RCC4-VHL cells under 20% O_2_ conditions (Fig. 3b).

Next, the RCC4-EV and RCC4-VHL cell growth rates were examined using the MTS assay (Fig. 3c). No significant difference in the cell growth rate of RCC4-VHL and RCC4-EV cells was found. However, RCC4-EV cells had a higher level of ATP, as compared with the RCC4-VHL cells (Fig. 3d).

### HIF-1 activation is required for resistance to propofol-induced cell death

**Figure 4:**
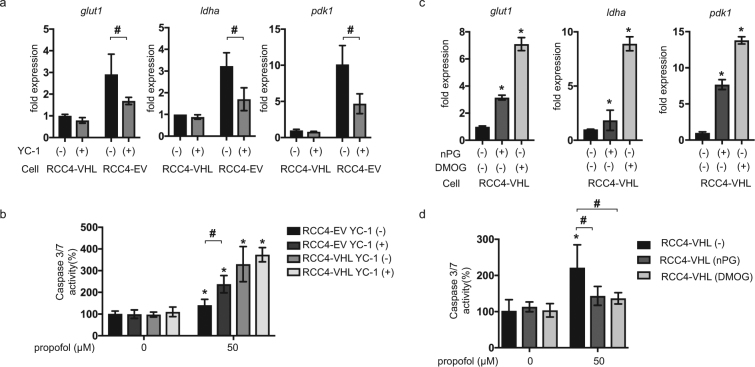
HIF-1 activation is required for RCC4-EV cells and sufficient for RCC4-VHL cells to confer resistance against propofol-induced cell death. (**a**) RCC4-EV and RCC4-VHL cells were incubated with (+) or without (−) 100 µM YC-1 for 24 h with 20% O_2_ prior to analysis of the indicated mRNA levels using *q*RT-PCR. Fold expression was calculated relative to the values measured for RCC4-VHL cells incubated with 20% O_2_. Data are expressed as the mean ± SD. Differences between results were evaluated by *t*-test; ^#^*p* < 0.05 for the indicated comparison. (**b**) Caspase 3/7 activity in RCC4-EV and RCC4-VHL cells (n = 3), incubated with or without 50 µM propofol for 6 h, with or without 100 µM YC-1 as indicated. Differences between results were evaluated by two-way ANOVA followed by Dunnett’s test for multiple comparisons; *p < 0.05, as compared to the control cells (no treatment); ^#^*p* < 0.05 for the indicated comparison. (**c**) RCC4-VHL cells were incubated with or without 100 µM nPG or 100 µM DMOG for 24 h, as indicated, prior to determination of the indicated mRNA levels by *q*RT-PCR. Differences between results were evaluated by one-way ANOVA followed by Dunnett’s test for multiple comparisons; *p < 0.05, as compared to the control cell population (no treatment). (**d**) Caspase 3/7 activity in RCC4-VHL cells (n = 3) that were exposed to the indicated treatments for 24 h prior to treatment with 50 µM propofol for 6 h. Differences between results were evaluated by two-way ANOVA followed by Dunnett’s test for multiple comparisons; *p < 0.05, as compared to the control cell population (no treatment); ^#^*p* < 0.05 for the indicated comparison.

Treatment of RCC4-EV cells with the HIF inhibitor, YC-1, reduced the expression of downstream genes such as *glut1*, *ldha* and *pdk1* in RCC4-EV cells but not in RCC4-VHL cells (Fig. 4a). The YC-1 treatment also significantly increased caspase 3/7 activation in RCC4-EV cells (Fig. 4b) within 6 h of exposure to 50 µM propofol. In contrast, significant impact of YC-1 on caspase 3/7 activation was not observed in RCC4-VHL cells. Next, RCC4-VHL cells were exposed to the HIFα-hydroxylase inhibitors, nPG (100 µM) and DMOG (100 µM). These treatments increased the expression of *glut1*, *ldha* and *pdk1* in RCC4-VHL cells (Fig. 4c). The treatments also significantly suppressed the caspase 3/7 activation (Fig. 4d) that was induced by 50 µM propofol in RCC4-VHL cells. These findings indicated that HIF activation was required and sufficient for establishment of cell protection against propofol-induced toxicity.

### Oxygen metabolism in RCC4 cells

**Figure 5:**
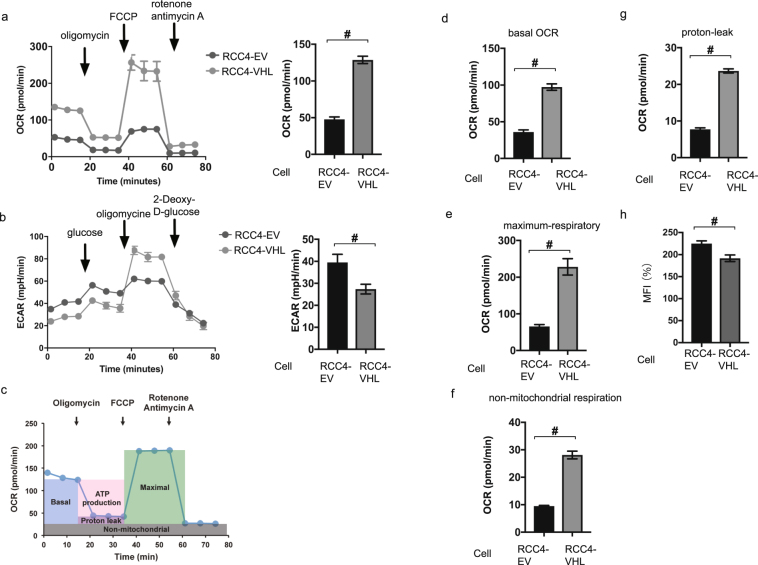
Reprogrammed oxygen metabolism in RCC4-EV cells. OCR (**a**) and ECAR (**b**) in the indicated cells under normoxic (20% O_2_) conditions. (**c**) Cell Mito Stress test™ profile of the key parameters of mitochondrial oxygen consumption rate (OCR). The values of basal OCR (**d**), maximal OCR (**e**), the non-mitochondrial respiration rate (**f**), and proton leakage (**g**) are also indicated for RCC4-EV and RCC4-VHL cells. (**h**) Equal numbers of RCC4 and RCC4- VHL cells were stained with MitoTracker™ Green FM and analyzed by flow cytometry to measure mitochondrial mass. Differences between results were evaluated by *t*-test ^#^*p* < 0.05 compared to the control cell population (group).

Next, we investigated oxygen utilization and glycolysis in RCC4-EV and RCC4-VHL cells using the assays measuring OCR by Cell Mito Stress Test (Fig. 5a) and ECAR by Glycolysis Stress Test (Fig. 5b). OCR was reduced and ECAR was increased in RCC4-EV cells. The mitochondrial basal OCR was significantly lower in RCC4-EV cells than in RCC4-VHL cells in the presence of 20% O_2_ (Fig. 5c,d). The significant differences were also detected in the maximum respiratory rates, non-mitochondrial respiration, and proton leak observed in the RCC4-EV and RCC4-VHL cells (Fig. 5e–g). In addition, significant differences in mitochondrial mass between RCC4-VHL cells and RCC4-EV cells was observed (Fig. 5h). Thus, we observed metabolic reprogramming from aerobic to anaerobic glucose metabolism in the RCC4-EV cells.

**Table 1:** Key Resources Table.

Reagents	Identifier	Source
Dulbecco’s modified Eagle’s medium	11965-092	Thermo Fisher Scientific, Waltham, MA, USA
RPMI medium	11875-093	
MitoTracker™ Green FM	M7514	
fetal bovine serum	SH30910	GE Healthcare, Little Chalfont, UK
Horseradish peroxidase-conjugated sheep anti-mouse IgG	NA931	
ECL prime enhanced chemiluminescence reagent	RPN2232	
penicillin-streptomycin	09367-34	Nacalai Tesque, Kyoto, Japan
D-(−)-mannitol	21303-32	
MgCl_2_	20909-42	
HEPES	17557-94	
EGTA	37346-05	
sodium pyruvate	06977-34	
L-(−)-malic acid	21030-44	
succinic acid disodium salt	32405-62	
L(+)-ascorbic acid sodium salt	03422-32	
sucrose	196-00015	Wako, Osaka, Japan
KH_2_PO_4_	169-04245	
albumin from bovine serum (BSA)	017-15141	
adenosine 5′-diphosphate, monopotassium salt (ADP)	303-50751	
N,N,N′,N′-tetramethyl-p-phenylenediamine (TMPD)	203-12821	
Dimethyloxaloylglycine (DMOG)	D3695	SIGMA, St Louis, MO, USA
n-propyl gallate (nPG)	P3130	
oligomycin	O4876	
carbonyl cyanide 4-(trifluoromethoxy) phenylhydrazone (FCCP)	C2920	
rotenone	R8875	
antimycin A	A8674	
CellTiter 96™ AQueous One Solution Cell Proliferation Assay	G3582	Promega, Madison, WI, USA
Caspase-Glo™ 9 Assay Kit	G8210	
Apo-ONE™ Homogeneous Caspase-3/7 Assay Kit	G7792	
CellTiter-Glo™ luminescent cell viability assay kit	G7570	
RNeasy™ Mini Kit	74104	Qiagen, Hilden, Germany
QuantiTect™ Reverse Transcription Kit	205311	
Rotor-Gene™ SYBR Green PCR Kit	204074	
anti-human HIF-1α antibody Clone 54/HIF-1α	610959	BD Biosciences, San Jose, CA, USA
HIF-1β/ARNT (D28F3) XP rabbit monoclonal antibody	5537	Cell Signaling Technology, Danvers, MA, USA
Complete Protease Inhibitor	4693116001	Roche Diagnostics, Tokyo, Japan
Annexin V-FITC Apoptosis Detection Kit	K101	BioVision, Milpitas, CA, USA
Seahorse XF Plasma Membrane Permeabilizer (PMP)	102504-100	Agilent Technologies, Santa Clara, CA, USA
tetramethylhydroquinone (duroquinol)	T0822	Tokyo Chemical Industry, Tokyo, Japan
2′,7′-dichlorofluorescin diacetate (DCFH-DA)	D399	Molecular Probes, Eugene, OR, USA

The local anesthetic lidocaine induced cell death by targeting mitochondria ETC as well as propofol. To investigate the mode of targeting of propofol, we examined OCR, which depends on the activity of mitochondrial respiratory chain complexes I–IV in membrane-permeabilized and intact cells, using an extracellular flux analyzer (Supplementary Figure 1). Propofol suppressed ETC complex I, II and III-dependent OCR but lidocaine suppressed only complex I (Supplementary Figure 1c,d).

### RNA-Seq analysis of RCC4-EV and RCC4-VHL cells

**Figure 6:**
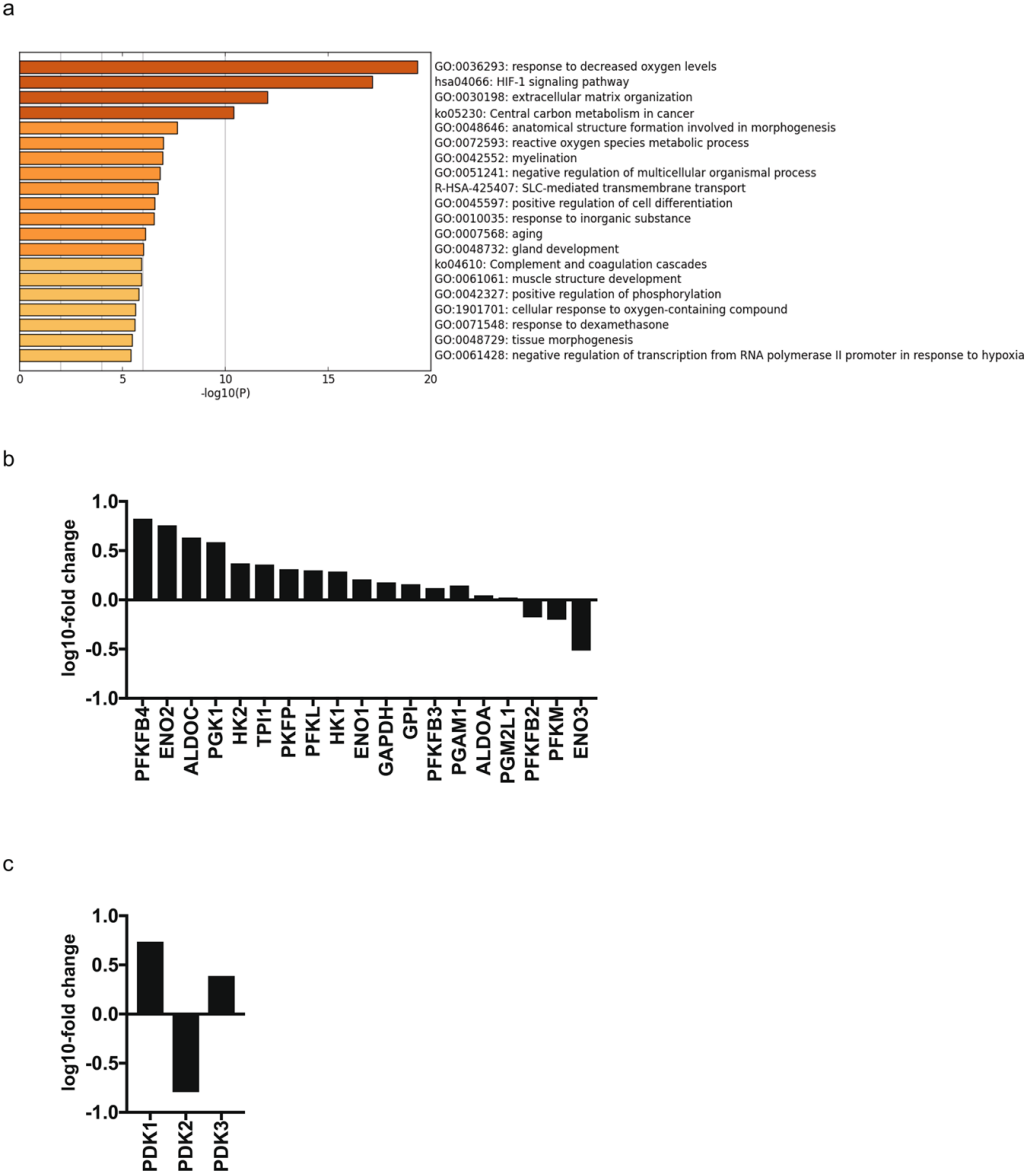
Gene set enrichment analysis of RCC4 cells. (**a**) A heatmap of enriched terms across the input gene lists, colored to indicate the *p* values. Cuffdiff and Metascape were used for this analysis. RNA-seq analysis of the expression levels of selected genes within (**b**) GO:0061621 (canonical glycolysis) and (**c**) GO:0004740 (pyruvate dehydrogenase (acetyl-transferring) kinase activity) in RCC4-EV and RCC4-VHL cells. The y axis indicates the ratio of the average FPKM values for RCC4-VHL cells.

We conducted a comprehensive gene expression analysis using RNA-Seq (Table S1) because a line of reports demonstrated that HIF-1 determines oxygen utilization and glucose metabolism^14–16^. Our RNA-Seq analysis also made it clear that there are differences in the cellular hypoxic pathway and HIF-1 signaling pathway in RCC4-EV and RCC4-VHL cells (Fig. 6a; Table S2). RNA-seq identified differences in the expression levels of selected genes within GO:0061621 (canonical glycolysis) (Fig. 6b) and GO: 0004740 (pyruvate dehydrogenase (acetyl-transferring) kinase activity) (Fig. 6c) in these cell lines. To confirm the experimental result of gene expression, we performed meta-analysis using FASTQ files deposited in the Sequence Read Archive (https://trace.ddbj.nig.ac.jp/dra/indexe.html) as SRR1554431, SRR1554986, SRR1554988 and SRR155499. Comparative analysis of gene expression differences between RCC4-EV cells and RCC-VHL cells demonstrated that our study was consistent with the data in SRA except for PGK1 (Supplementary Figure 2). This is because the expression intensity of PGK1 was too high and beyond the dynamic range of RNA-seq. Thus, the expression difference of PGK1 could not be calculated properly.

### ROS generation in RCC4-EV and RCC4-VHL cells in response to propofol treatment

**Figure 7:**
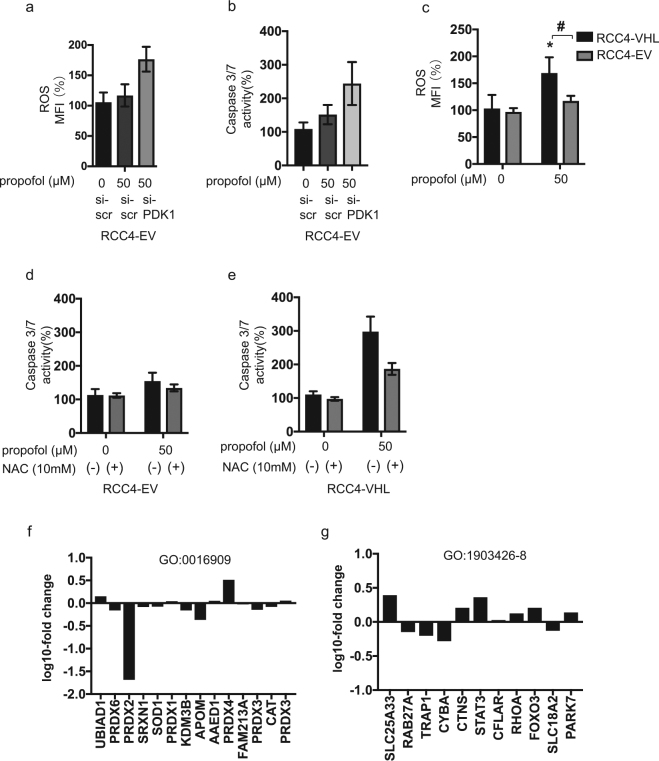
ROS production in response to propofol treatment is reduced in RCC4-EV cells. (**a** and **b**) RCC4-EV cells were transfected with small interfering RNA (siRNA) targeting pyruvate dehydrogenase kinases 1 (PDK-1) or a negative control (scr). Cells were exposed to 50 µM propofol and ROS generation (**a**) and caspase 3/7 (**b**) were assayed. (**c**) ROS generation in RCC4-VHL cells and RCC4-EV cells exposed to 50 µM propofol (n = 3) were assayed. (**d** and **e**) RCC4-EV (d) and RCC4-VHL (**e**) cells were exposed to 50 µM propofol for 6 h with or without 10 mM NAC treatment. Caspase 3/7 activity of the cells were assayed. Differences were evaluated by two-way ANOVA followed by Dunnett’s test for multiple comparisons; *p < 0.05, as compared to the control cells (no treatment); ^#^*p* < 0.05 for the indicated comparison. (**f** and **g**) RNA-seq analysis of the expression levels of selected genes within (**f**) GO:0016909 (antioxidant activity) and (**g**) GO:1903426-8 (regulation of reactive oxygen species biosynthetic process) in RCC4-EV and RCC4-VHL cells. The y axis indicates the ratio of the average FPKM values for RCC4-VHL cells.

Next, we investigated that impact of gene silencing of PDK1 on ROS generation and caspase 3/7 activity in response to propofol treatment in RCC4-EV cells. Lack of PDK1 gene expression induced ROS generation and caspase 3/7 activation (Fig. 7a,b). We found that ROS generation played a critical role in propofol-induced cell death. Here, we compared ROS levels in RCC4-VHL and RCC4-EV cells exposed to 50 µM propofol. ROS generation in response to 50 µM propofol treatment was observed in RCC4-VHL cells, but not in RCC4-EV cells (Fig. 7c). Moreover, treatment with the antioxidant NAC suppressed 50 µM propofol induced caspase 3/7 activation exclusively in RCC4-VHL cells (Fig. 7d,e). Next, we investigated the expression of genes which related to generation and scavenging ROS by RNA-Seq analysis. The expression levels of selected genes within GO:0016909 (antioxidant activity) (Fig. 7f) and GO:1903426-8 (regulation of reactive oxygen species biosynthetic process) (Fig. 7g) were investigated in these cell lines.

### Effect of exogenous HIF-1 activation on propofol toxicity in neuronal SH-SY5Y cells

**Figure 8:**
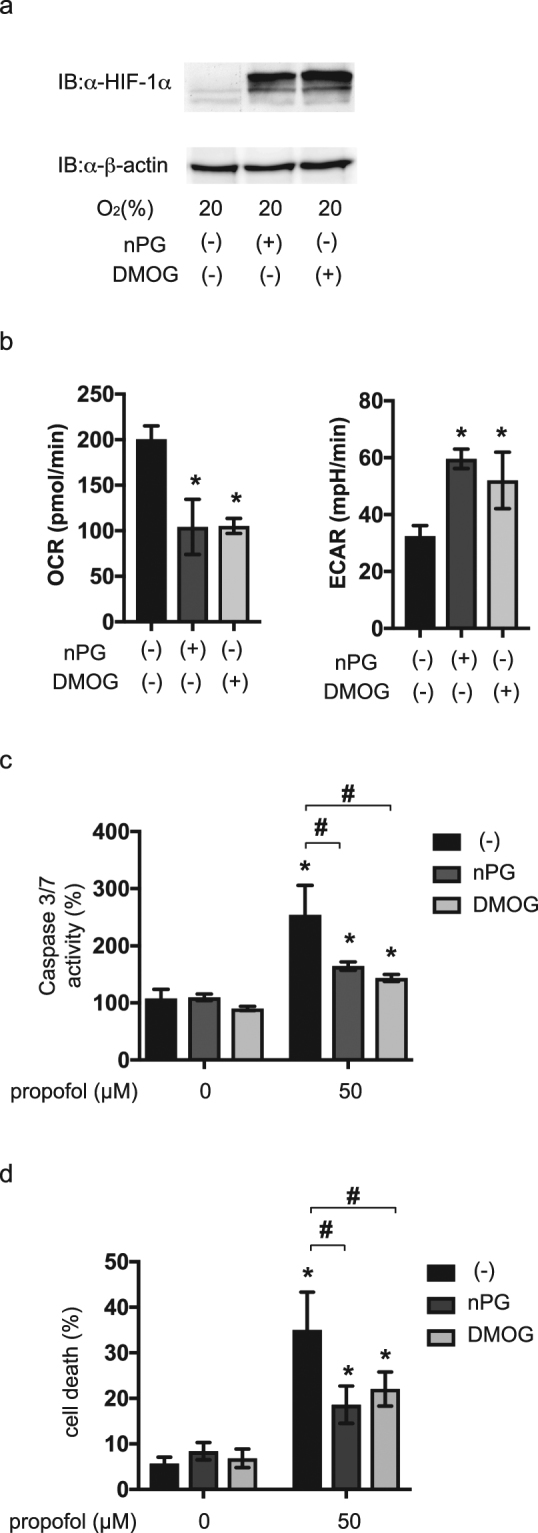
Forced HIF-1 activation is sufficient to resist propofol toxicity in SH-SY5Y neuroblastoma cells. (**a**) SH-SY5Y cells were incubated with (+) or without (−) 200 µM nPG or 1 mM DMOG for 4 h. 35 µg of whole-cell lysates were immunoblotted (IB) using primary antibodies raised against the indicated proteins. (**b**) OCR and ECAR were measured in SH-SY5Y cells exposed to 100 µM nPG or 100 µM DMOG for 4 h. (**c** and **d**) SH-SY5Y cells were exposed to 100 µM nPG and 100 µM DMOG for 4 h and then exposed to 50 µM propofol for 6 h. Graphic depictions of caspase-3/7 activity (n = 3) (**c**) and cell death (n = 3) (**d**). Differences were evaluated by two-way ANOVA followed by Dunnett’s test for multiple comparisons; *p < 0.05, as compared to the control cells (no treatment); ^#^*p* < 0.05 for the indicated comparison.

Finally, effect of exogenous HIF-1 activation on propofol-induced toxicity was examined in a different cell-type from RCC4 cells. Human neuroblastoma SH-SY5Y cells were treated with 100 µM nPG or 100 µM DMOG in the presence of 20% O_2_. The treatments induced expression of HIF-1α protein (Fig. 8a), suppressed OCR, and increased ECAR (Fig. 8b) in these cells. Consistent with our findings in RCC4-VHL cells, treatment with nPG or DMOG conferred resistance to the caspase 3/7 activation induced by 50 µM propofol (Fig. 8c) and cell death (Fig. 8d).

## Discussion

In this study, we demonstrated that activation of HIF-1 by genetic or pharmacological means induced metabolic reprogramming and attenuated the ROS generation and cell death induced by a clinical relevant concentration of propofol; these effects were observed in both the established renal carcinoma RCC4 cell line and in the neuroblastoma SH-SY5Y cell line.

In RCC4 cells, the VHL gene is ablated^10^. Because VHL is an essential component of the E3 ubiquitin ligase, VHL regulates HIFα subunit protein expression^9,20^. HIF-1 is therefore activated in RCC4-EV cells under both 20% and 1% O_2_ conditions. As demonstrated by the present RNA-Seq gene expression analysis, enrichment analysis, and *q*RT-PCR study, canonical glycolysis and the HIF-1-dependent pathway were activated in RCC4-EV cells. Thus the global gene expression analysis demonstrates change of metabolic mode from OXPHOS to glycolysis in RCC4-EV cells.

O_2_ is primarily required for OXPHOS within cells. The maximal respiration rates in RCC4-EV cells were downregulated, as compared to the rates observed in RCC4-VHL cells. This indicated that mitochondrial electron transport was significantly inhibited in RCC4-EV, as compared to RCC4-VHL cells. Substrate availability is also a critical regulator of OXPHOS. While the most critical substrate for OXPHOS is O_2_, acetyl-CoA is another critical regulator of this process. The conversion of pyruvate to acetyl-CoA thus represents a critical regulatory point in cellular energy metabolism^17,21^. Pyruvate dehydrogenase is regulated by PDK-mediated phosphorylation of its E1 subunit. PDK1 is downstream of HIF-1 and it negatively regulates mitochondrial function by reducing pyruvate entry into the tricarboxylic acid cycle. The present study found that PDK1 mRNA expression increased in RCC4-EV cells to a greater extent than in RCC4-VHL or SH-SY5Y cells following treatment with HIFα-hydroxylase inhibitors. Suppression of PDK1 expression by siRNA increased ROS in response to propofol treatment. Proton leak, as defined by the mitochondrial respiration rate in the presence of oligomycin, was lower in RCC4-EV cells than in RCC4-VHL cells. Mitochondrial superoxide production is highly dependent on the membrane potential^22,23^ and proton leak pathways may therefore minimize oxidative damage by reducing this potential and thus suppressing superoxide production. Together with metabolic reprogramming, HIF-1dependent gene expression change contributes to change of mode of electric transport in mitochondria.

Intriguingly, RCC4-EV cells had a higher intracellular ATP concentration than RCC4-VHL cells. This was consistent with the more active ECAR in RCC4-EV cells. ECAR provides a surrogate marker of glycolysis^21^, and the higher ECAR in RCC4-EV cells therefore indicated that this ATP was derived from glycolysis. We recently demonstrated that clinically relevant doses of propofol suppressed mitochondrial electron transport in SH-SY5Y cells in a dose- and time-dependent manner^5^. Consistent with this conclusion, mitochondrial DNA-deficient cells were shown to be resistant to propofol-induced toxicity^5^. The reduction of O_2_ to H_2_O by complex IV is not completely efficient. If electron transfer to O_2_ occurs at complexes I or III, ROS generation occurs and these free radicals can oxidize cellular proteins, lipids, and nucleic acids. The ROS plays a critical role in propofol-induced cell death. In fact, the treatment with the antioxidant NAC reduced propofol-induced caspase 3/7 activation. Propofol induced significantly less ROS production in RCC4-EV cells than in RCC4-VHL cells. Propofol has also been shown to suppress the activity of complexes I, II and III, and to reduce mitochondrial oxygen consumption^5^. The RNA-Seq analysis indicated that HIF-1 activation on RCC4-EV cells induced gene expression which facilitate glycolysis but not significantly induced gene sets of peroxidase activity or reactive oxygen species metabolic process. Thus, HIF target gene activation is upstream of mitochondrial function and can alter mitochondrial activity.

Preclinical studies in animal models have predicted that systemic HIF activation has the potential to alter glucose, fat, and mitochondrial metabolism^24^. Indeed, a series of HIFα-hydroxylase inhibitors are currently undergoing evaluation in clinical anemia trials^25,26^. Thus, in addition to nPG and DMOG, these HIFα-hydroxylase inhibitors could modulate mitochondrial metabolism and may prevent the cell death that occurs during propofol infusion syndrome. The findings of the present study warrant a preclinical trial of these inhibitors for the treatment of this syndrome in an animal model.

In conclusion, VHL deletion or exposure to small-molecule HIFα-hydroxylase inhibitors activates HIF-1 and cellular metabolic reprogramming and oxygen utilization of mitochondria. The HIF-1 activation suppresses ROS generation and confers resistance to propofol toxicity.

## Materials and Methods

### Cell culture and reagents

Renal cell carcinoma cell lines were stably transfected with pcDNA3-VHL (RCC4-VHL) or empty vector (RCC4-EV)^6^. These cells lines were maintained in Dulbecco’s modified Eagle’s medium supplemented with 10% fetal bovine serum, 100 U/ml penicillin, and 0.1 mg/ml streptomycin. The human neuroblastoma SH-SY5Y cells were maintained in RPMI medium supplemented with 10% fetal bovine serum, 100 U/ml penicillin, and 0.1 mg/ml streptomycin^6,27,28^. Purified mouse anti-human HIF-1α antibody Clone 54/HIF-1α was purchased from BD Biosciences (San Jose, CA). HIF-1β/ARNT (D28F3) XP rabbit monoclonal antibody was from Cell Signaling Technology (Danvers, MA). Dimethyloxaloylglycine (DMOG) and n-propyl gallate (nPG) the anti-β-actin antibody were obtained from Sigma. A list of reagents used in this study is provided in Table 1.

### Cell growth assay

Cell growth was assessed using a CellTiter 96™ AQueous One Solution Cell Proliferation Assay (Promega, Madison, WI, USA)^6,28^. This assay measured the reduction of the tetrazolium compound, MTS (3-[4,5-dimethyl-2-yl]-5-[3-carboxymethoxyphenyl]-2-[4-sulfophenyl]-2H-tetrazolium, inner salt). Briefly, cells were seeded into 96-well plates (3 × 10^3^ cells/well) and cultivated for 24, 48 and 72 h. 20 μl of CellTiter 96 AQueous One Solution™ Reagent was added to each well and the plates were incubated at 37 °C for 1 h prior to measuring the absorbance of each sample using an iMark™ Microplate Reader (BIO-RAD, Hercules, CA, USA) at a wavelength of 490 nm. Cell viability was then calculated by comparing the absorbance of the treated cells with that of the control cells (RCC4-VHL cells at 24 h incubation), which was defined as 100%. All samples were assayed in triplicate or quadruplicate for each experiment.

### Caspase activity assays

The levels of caspase 9 and caspase 3/7 activity were assessed using a Caspase-Glo™ 9 Assay Kit (Promega) and an Apo-ONE™ Homogeneous Caspase-3/7 Assay Kit (Promega), respectively, according to the manufacturer’s protocols^6,28^. Briefly, cells were seeded into 96-well plates (2 × 10^4^ cells/well) and incubated overnight. The following day, cells were treated with the indicated concentrations of the appropriate drug(s) for varying lengths of time. After treatment, 100 μl of Apo-ONE Caspase-3/7 Reagent™ was added to each well. Cells were incubated at room temperature for 1 h and the luminescence of each well was measured using an EnSpire™ Multimode Plate Reader (PerkinElmer, Waltham, MA, USA). Caspase activity was then calculated by comparing the levels of luminescence of the treated cells with that of the control cells (incubated without drugs), which was defined as 100%. Assays were performed in triplicate at least twice. Data were expressed as means ± the standard deviation (SD).

### Immunoblot assays

Whole-cell lysates were prepared as described previously^6,27,29^. In brief, these were prepared using ice-cold lysis buffer containing 0.1% sodium dodecyl sulfate, 1% Nonidet P-40, 5 mM ethylenediaminetetraacetic acid, 150 mM NaCl, 50 mM Tris-Cl (pH 8.0), 2 mM dithiothreitol, 1 mM sodium orthovanadate, and Complete Protease Inhibitor™ (Roche Diagnostics, Tokyo, Japan). Samples were centrifuged at 10,000 × g to sediment the cell debris, and the supernatant was used for subsequent immunoblotting experiments. For HIF-1α and HIF-1β determinations, 35 µg of protein was fractionated by sodium dodecyl sulfate-polyacrylamide gel electrophoresis (7.5% gel), transferred to membranes, and immunoblotted using the indicated primary antibodies at a dilution of 1:500. Horseradish peroxidase-conjugated sheep anti-mouse IgG (GE Healthcare, Piscataway, NJ) was used as the secondary antibody, at a dilution of 1:2,000. The signal was developed using enhanced chemiluminescence reagent (GE Healthcare). Experiments were repeated at least three times and representative blots are shown.

### Analysis of cell death

Cell death was investigated using a previously described protocol^6,28^. Briefly, apoptosis was measured using an Annexin V-FITC Apoptosis Detection Kit (BioVision, Milpitas, CA, USA), according to the manufacturer’s instructions. For these analyses, cells were seeded into 6-well plates (3 × 10^5^ cells/well) and incubated overnight. The following day, cells were treated with the indicated concentrations of the appropriate drug(s) for varying lengths of time prior to harvesting by centrifugation at 1,200 rpm for 3 min. The culture supernatants were discarded and the pellets were resuspended in a mixture of 500 μl binding buffer, 5 μl annexin V-fluorescein isothiocyanate (FITC), and 5 μl propidium iodide (PI; 50 μg/ml) for 5 min at room temperature in the dark prior to analysis using a FACSCalibur flow cytometer (BD Biosciences, San Jose, CA, USA) equipped with CellQuest Pro™ software. Data were evaluated using FlowJo™ version 7.6.3 software (TreeStar, Ashland, OR, USA), exported to Excel spreadsheets, and subsequently analyzed using the statistical application, Prism7™.

### ATP assay

The CellTiter-Glo™ luminescent cell viability assay kit (Promega, Madison, WI) was used to evaluate the intracellular ATP content^6^. Briefly, cells were seeded in 96-well plates (3 × 10^3^ cells/well) and allowed to grow for 24, 48 and 72 h. CellTiter-Glo reagent (50 μl) was then added directly into each well and incubated for 10 min prior to reading the plate using an EnSpire™ Multimode Plate Reader (PerkinElmer, Waltham, MA, USA). This detected the luminescence generated by the luciferase-catalyzed reaction between luciferin and ATP. Assays were performed in triplicate at least twice. The ATP content was then calculated by comparing the luminescence levels of RCC4-VHL cells with that of RCC4-EV cells, which was defined as 100%. Data were expressed as the mean ± SD.

### Measurement of cellular oxygen consumption and extracellular acidification

The cellular oxygen consumption rate (OCR) and extracellular acidification rate (ECAR) were respectively detected by the XF Cell Mito Stress Test™ and XF Glycolysis Stress Test™ using a XFp Extracellular Flux Analyzer™ (Agilent Technologies, Santa Clara, CA)^6^. RCC4-EV and RCC4-VHL cells were seeded onto the XFp Cell Culture microplate at a density of 1 × 10^4^ cells/well. The XF Cell Mito Stress Test™ for OCR was assessed in glucose-containing XF base medium, according to the manufacturer’s instructions. The sensor cartridge for the XFp analyzer was hydrated at 37 °C in a non-CO_2_ incubator one day before the experiment. For the OCR assay, the injection port A on the sensor cartridge was loaded with oligomycin (a complex V inhibitor, final concentration 1 µM), carbonyl cyanide-p-trifluoromethoxyphenylhydrazone (FCCP; an uncoupling agent, final concentration 2 µM) was loaded to port B, and rotenone/antimycin A (inhibitors of complexes I and III, final concentration 0.5 µM each) was loaded to port C. During the sensor calibration, cells were incubated at 37 °C in the non-CO_2_ incubator in 180 μl assay medium (XF Base Medium, 25 mM glucose, 1 mM pyruvate, and 2 mM l-glutamine, pH 7.4). The plate was immediately placed onto the calibrated XFp Extracellular Flux Analyzer for the assay.

The minimum OCR measured after rotenone/antimycin A injection was considered to represent the non-mitochondrial respiration rate. The basal OCR was calculated by subtracting the non-mitochondrial respiration rate from the last OCR measurement before oligomycin injection. The maximal OCR was calculated by subtracting the non-mitochondrial respiration rate from the maximum OCR measurement after FCCP injection. The proton leakage was calculated by subtracting the non-mitochondrial respiration rate from the minimum OCR measured after oligomycin injection.

For XF Glycolysis Stress Test™ for the ECAR, injection port A on the sensor cartridge was loaded with glucose (final concentration 10 mM), 2-Deoxy-D-glucose (final concentration 50 mM) loaded to portB and olygomycin (final concentration 1 µM) was loaded to port C. During the sensor calibration, cells were incubated at 37 °C in 180 μl assay medium (XF Base Medium and 2 mM l-glutamine, pH 7.4) in the non-CO_2_ incubator. The plate was immediately placed into the calibrated XFp Extracellular Flux Analyzer for the assay.

### Measurement of oxygen consumption in permeabilized cells

The activity of individual respiratory chain complexes was evaluated in permeabilized cells^30,31^. Briefly, cells were washed with mitochondrial assay solution (MAS) buffer (220 mM mannitol, 70 mM sucrose, 10 mM KH_2_PO_4_, 5 mM MgCl_2_, 2 mM HEPES, 1 mM EGTA, 0.2% fatty acid-free bovine albumin, adjusted to pH 7.2 with KOH), and the medium was replaced with MAS buffer supplemented with 10 mM pyruvate, 1 mM malate, 4 mM ADP, and 1 nM plasma membrane permeabilizer™. The cells were then loaded into the XFp analyzer to measure respiration rates using cycles of 30 s mixing/30 s waiting/2 min measurement. Protocol A: After the measurement of pyruvate-driven respiration, rotenone (final concentration 2 µM) was injected through port A to halt the complex I-mediated respiratory activity. Next, succinate (10 mM) was injected through port B to donate electrons at complex II, bypassing complex I inhibition. The addition of antimycin A (2 µM) via port C inhibited complex III, and N,N,N′,N′-tetramethyl-*p*-phenylenediamine (TMPD 0.1 mM), combined with ascorbate (10 mM), was subsequently injected through port D to measure complex IV activity. Protocol B: As an alternative approach, cells were initially supplemented with pyruvate to measure complex I activity. After injection of rotenone, duroquinol was injected to stimulate complex III-mediated respiration.

### Mitochondrial mass assay

Mitochondrial mass was measured by staining cells with MitoTracker™ Green FM at 37 °C for 15 min in PBS containing 5% FBS. Stained cells were filtered and analyzed immediately in a FACScan flow cytometer (BD Bioscience). Mean fluorescence intensity was analyzed using CellQuest software (BD Bioscience).

### Semi-quantitative real-time reverse transcriptase-polymerase chain reaction analysis (*q*RT-PCR)

Total RNA was extracted from cells using the RNeasy™ Mini Kit (Qiagen, Hilden, Germany), according to the manufacturer’s instructions^6^. First-strand synthesis and RT-PCR were performed using the QuantiTect™ Reverse Transcription Kit (Qiagen) and Rotor-Gene™ SYBR Green PCR Kit (Qiagen), according to the manufacturer’s protocol. Amplification and detection were performed using Rotor-Gene™ Q (Qiagen). PCR primers were purchased from Qiagen. The change in expression of each target mRNA was calculated relative to the level of 18S rRNA.

### Measurement of ROS generation

ROS generation was detected using 2′,7′-dichlorofluorescin diacetate (DCFH-DA) (Molecular Probes, Eugene, OR). Briefly, cells were cultured in 35-mm diameter glass-bottomed culture dishes (MatTek, Ashland, MA) and incubated with 10 µM DCFH-DA for 10 min at 37 °C in serum-free Dulbecco’s modified Eagle’s medium. The cells were then washed twice with Dulbecco’s phosphate-buffered saline and analyzed using a flow cytometer (Becton Dickinson, San Jose, CA)^32^. Mean fluorescence intensity was analyzed using CellQuest software (Becton Dickinson).

### RNA-Seq protocol

Total RNA was extracted from RCC4-EV and RCC4-VHL cells using RNeasy Mini Kit (Qiagen). RNA-Seq libraries were prepared using TruSeq Stranded mRNA Sample Prep Kit for Illumina multiplexed sequencing (Illumina, San Diego, CA, USA). TruSeq stranded mRNA library preparation kit was used to generate poly (A) RNA libraries for RNA obtained from normal skin samples (Illumina, San Diego, CA, USA). Libraries were sequenced (100 bp, paired-end) on the Illumina HiSeq2500 platform. The sequence data (FASTQ files)were deposited in the DDBJ Sequence Read Archive under accession numbers DRR100656 and DRR100657, respectively.

### RNA-Seq data analysis

FASTQ files for RCC4-EV cells (DRR100656) and RCC4-VHL cells (DRR100657) were evaluated by FastQC (http://www.bioinformatics.babraham.ac.uk/projects/fastqc/) after the trimming process by FASTX-Toolkit v0.0.14 (http://hannonlab.cshl.edu/fastx_toolkit/). The human reference sequence file (hs37d5.fa) was downloaded from the 1000 Genomes Project ftp site (ftp://ftp.1000genomes.ebi.ac.uk/vol1/ftp/technical/reference/phase2_reference_assembly_sequence/), and the annotated general feature format (gff) file was downloaded from the Illumina iGenome ftp site (ftp://igenome:G3nom3s4u@ussd-ftp.illumina.com/Homo_sapiens/NCBI/build37.2/). The human genome index was constructed with bowtie-build in Bowtie v.2.2.9^33^. The FASTQ files were aligned to the reference genomic sequence by TopHat v.2.1.1, with default parameters^34^. Bowtie2 v2.2.9 and Samtools v.1.3.1 were used within the TopHat program^35^. The estimated transcript abundance was calculated, and the count values were normalized to the upper quartile of the fragments per kilobase of transcript per million mapped reads (FPKM) using Cufflinks (Cuffdiff) v2.1.1^36^.

Metascape (http://metascape.org/) was used for the gene set enrichment analysis^37^. A gene list for Metascape analysis was generated from the Cuffdiff output, where 235 genes were identified as ‘significantly differentially expressed’ (*p* < 0.05; Table S1).

Genes with the gene ontology (GO) annotations of ‘canonical glycolysis’ (GO:0061621), ‘pyruvate dehydrogenase (acetyl-transferring) kinase activity’ (GO:0004740), ‘antioxidant activity’ (GO:0016909) and ‘regulation of reactive oxygen species biosynthetic process’ (GO:1903426-8) were extracted using Ensembl Biomart^38^ and sorted by the common logarithms of ([FPKM of RCC4-EV] + 1)/([FPKM of RCC4-VHL] + 1), calculated from the same Cuffdiff output file (Table S1). We added 1 to the FPKM values because it is not possible to calculate the logarithm of 0. A histogram was generated using TIBCO Spotfire Desktop v7.6.0 with the “Better World” program license (TIBCO Spotfire, Inc., Palo Alto, CA, USA) (http://spotfire.tibco.com/better-world-donation-program/).

### Statistical analysis

All experiments were repeated at least twice and each sample was evaluated in triplicate. Data are expressed as the mean ± SD. Differences between results were evaluated by one-way analysis of variance (ANOVA), two-way ANOVA followed by Dunnett’s test for multiple comparisons, or *t*-test using Prism7™ (GraphPad Software, Inc. La Jolla, CA). *P*-values < 0.05 were considered statistically significant^6,28^.

## Electronic supplementary material

Supplementary Information
