# Supplementary material for: Suppression of mitochondrial oxygen metabolism mediated by the transcription factor HIF-1 alleviates propofol-induced cell toxicity
Source: Sci Rep. 2018 Jun 12;8:8987. doi: 10.1038/s41598-018-27220-8 (PMC5997661; doi:10.1038/s41598-018-27220-8)
Supplement: Supplementary file 1 — Supplementary Information [file 41598_2018_27220_MOESM1_ESM.pdf]

Suppression of mitochondrial oxygen metabolism mediated by the transcription factor  
HIF-1 alleviates propofol-induced cell toxicity

Chisato Sumi<sup>1,2</sup>, Akihisa Okamoto<sup>2</sup>, Hiromasa Tanaka<sup>2</sup>, Munenori Kusunoki<sup>1,2</sup>,  
Tomohiro Shoji<sup>1,2</sup>, Takeo Uba<sup>1,2</sup>, Takehiko Adachi<sup>3</sup>, Teppei Iwai<sup>2</sup>, Kenichiro Nishi<sup>2</sup>,  
Hiroshi Harada<sup>4,5</sup>, Hidemasa Bono<sup>6</sup>, Yoshiyuki Matsuo<sup>2</sup>, and Kiichi Hirota<sup>2</sup>\*

<sup>1</sup>Department of Anesthesiology, Kansai Medical University, Hirakata, Japan

<sup>2</sup>Department of Human Stress Response Science, Institute of Biomedical  
Science, Kansai Medical University, Hirakata, Japan

<sup>3</sup>Department of Anesthesiology, Tazuke Kofukai Medical Research Institute,  
Osaka, Japan

<sup>4</sup>Laboratory of Cancer Cell Biology, Radiation Biology Center, Kyoto  
University, Kyoto, Japan

<sup>5</sup>Precursory Research for Embryonic Science and Technology (PRESTO),  
Japan Science and Technology Agency (JST), Saitama, Japan

<sup>6</sup>Database Center for Life Science (DBCLS), Research Organization of  
Information and Systems (ROIS), Mishima, Japan

## Supplementary Informations

Table S1 | Results of Data analysis of RNA-Seq

File is deposited at <https://doi.org/10.6084/m9.figshare.5353462>

FASTQ files for RCC4-EV cells (DRR100656) and RCC4-VHL cells (DRR100657) were evaluated by FastQC (<http://www.bioinformatics.babraham.ac.uk/projects/fastqc/>) after the trimming process by FASTX-Toolkit v0.0.14 ([http://hannonlab.cshl.edu/fastx\\_toolkit/](http://hannonlab.cshl.edu/fastx_toolkit/)). The human reference sequence file (hs37d5.fa) was downloaded from the 1000 genome ftp site ([ftp://ftp.1000genomes.ebi.ac.uk/vol1/ftp/technical/reference/phase2\\_reference\\_assembly\\_sequence/](ftp://ftp.1000genomes.ebi.ac.uk/vol1/ftp/technical/reference/phase2_reference_assembly_sequence/)), and the annotated general feature format (gff) file was downloaded from the Illumina iGenome ftp site ([ftp://igenome:G3nom3s4u@ussd-ftp.illumina.com/Homo\\_sapiens/NCBI/build37.2/](ftp://igenome:G3nom3s4u@ussd-ftp.illumina.com/Homo_sapiens/NCBI/build37.2/)). The human genome index was constructed with bowtie-build in Bowtie v.2.2.9. The fastq files were aligned to the reference genomic sequence by TopHat v.2.1.1 with default parameters. Bowtie2 v2.2.9 and Samtools v.1.3.1 was used with the TopHat program<sup>47</sup>. Estimation of transcript abundance was calculated, and the count values were normalized to the upper quartile of the fragments per kilobase of transcript per million fragments mapped reads (FPKM) using Cufflinks (cuffdiff) v2.1.1. cuffdiff output (gene\_exp. diff) was presented (gene\_exp.diff.txt). Sample\_1 "Ir5163" represents the sample from for RCC4-EV cells and sample\_2 "Ir5165" represents the sample from for RCC4-VHL cells.

Table S2 | Result of analysis with Metascape (Gene List)

File is deposited <https://doi.org/10.6084/m9.figshare.5353480>

Metascape (<http://metascape.org/>) was used for the gene set enrichment analysis <sup>1</sup>. A gene list for metascape analysis was generated from the output of cuffdiff program, in which 234 genes judged as 'significantly differentially expressed' ( $p<0.05$ ) in cuffdiff output (gene\_exp.diff) were indicated.

- 1      Tripathi, S. *et al.* Meta- and Orthogonal Integration of Influenza "OMICs" Data Defines a Role for UBR4 in Virus Budding. *Cell Host Microbe* **18**, 723-735, doi:10.1016/j.chom.2015.11.002 (2015).

Table S3 | Comparative meta-analysis of gene expression of GO:0061621 between public data in the Sequence Read Archive and data of this study

Fold gene expression of GO:0061621 of FASTQ files deposited in the Sequence Read Archive (<https://trace.ddbj.nig.ac.jp/dra/indexe.html>) as SRR1554431, SRR1554986, SRR1554988 and SRR155499 and FATSQ files from this study were calculated.

| gene   | This Study | Public Data |
|--------|------------|-------------|
| PFKFB4 | 0.83       | 0.65        |
| ENO2   | 0.76       | 0.59        |
| ALDOC  | 0.63       | 0.42        |
| PGK1   | 0.59       | -1.50       |
| HK2    | 0.37       | 0.39        |
| TPI1   | 0.36       | 0.32        |
| PKFP   | 0.31       | 0.00        |
| PFKL   | 0.30       | 0.16        |
| HK1    | 0.29       | 0.32        |
| ENO1   | 0.21       | 0.08        |
| GAPDH  | 0.18       | 0.17        |
| GPI    | 0.16       | 0.20        |
| PFKFB3 | 0.12       | 0.12        |
| PGAM1  | 0.15       | 0.10        |
| ALDOA  | 0.05       | 0.37        |
| PGM2L1 | 0.02       | 0.00        |
| PFKFB2 | -0.18      | -0.17       |
| PFKM   | -0.20      | -0.11       |
| ENO3   | -0.51      | -0.40       |

Supplementary figure 1

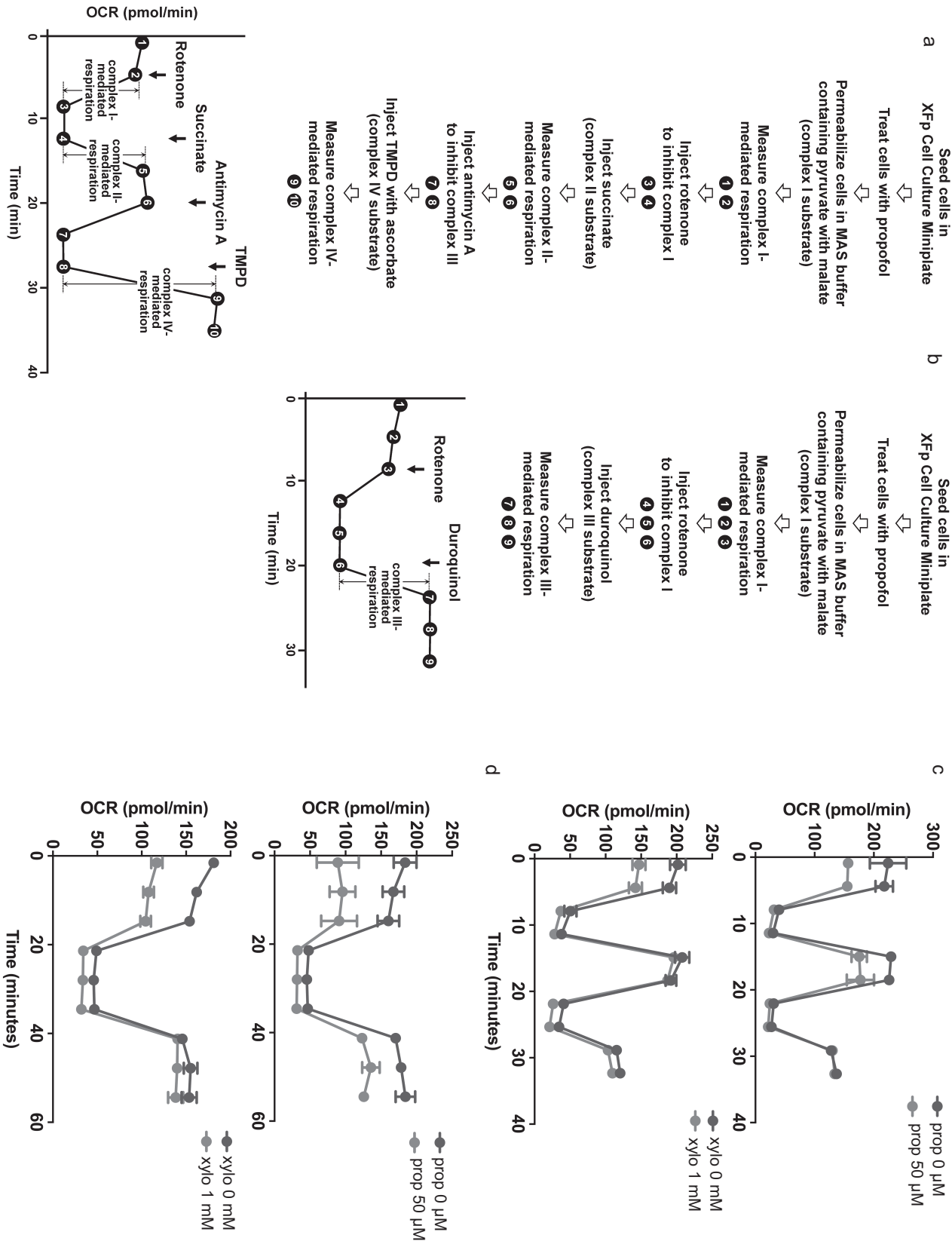

## Supplementary Figure 1 | Effects of propofol on OCR driven by each complex of the mitochondrial ETC

Representative OCR traces of mitochondrial respiration using protocol A

(Supplementary figures 1a and 1c) and protocol B (Supplementary figures 1b and 1d).

Mitochondrial ETC-mediated OCR, driven by complexes I, II, III, and IV were assayed using an extracellular flux analyzer. SH-SY5Y cells were exposed to 1mM lidocaine or 50  $\mu$ M propofol for 6 h and subjected to the assay.

Supplementary figure 2

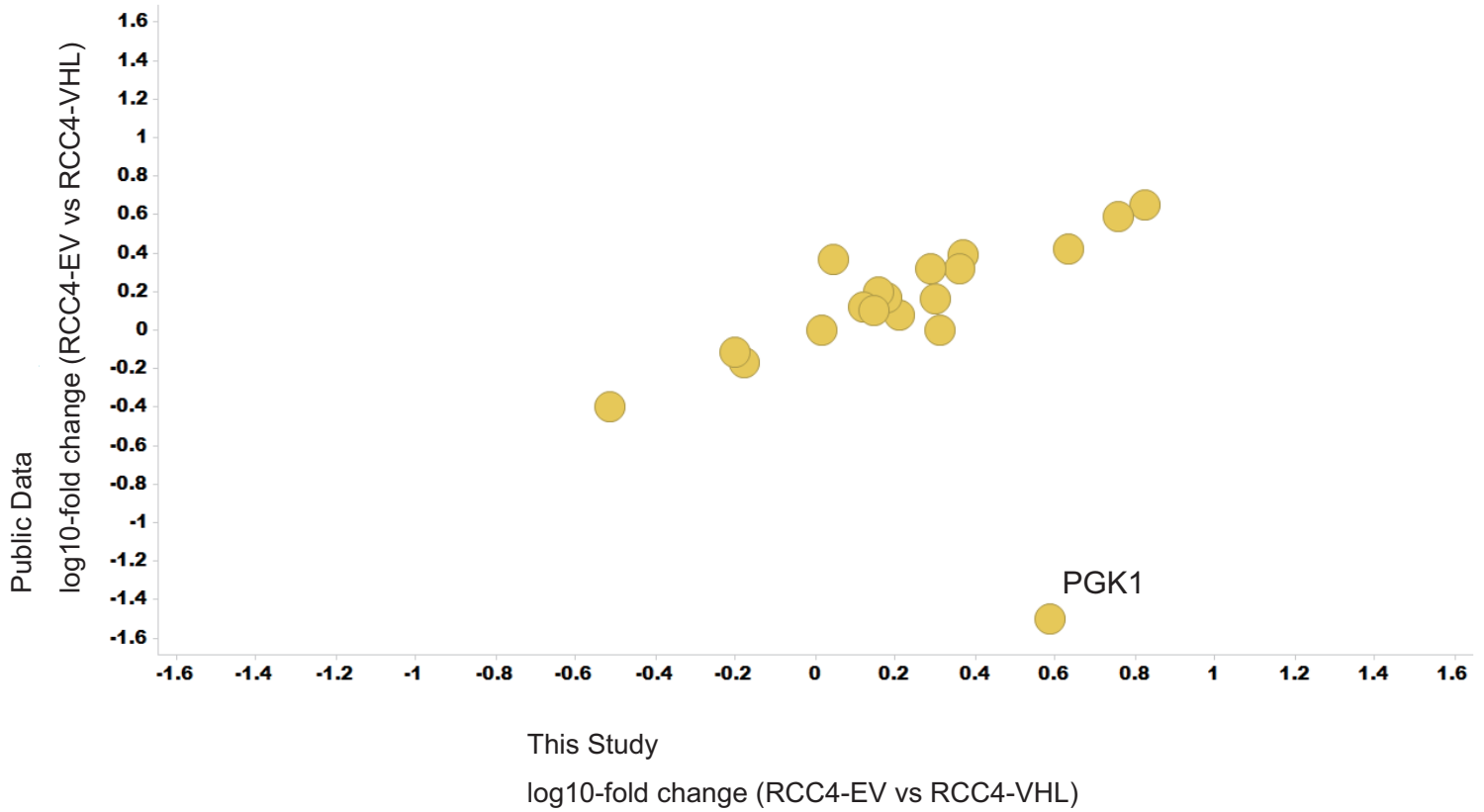

Supplementary figure 2 | Comparative meta-analysis of gene expression of GO:0061621 between public data in the Sequence Read Archive and data of this study

Fold gene expression (supplementary Table S3, RCC4-EV cells vs. RCC4-VHL cells) of GO:0061621 of FASTQ files deposited in the Sequence Read Archive (<https://trace.ddbj.nig.ac.jp/dra/indexe.html>) as SRR1554431, SRR1554986, SRR1554988 and SRR155499 and FATSQ files from this study.
